# Personalized virtual reality in hemodialysis patients: a multicenter pilot study

**DOI:** 10.1093/ckj/sfaf367

**Published:** 2025-12-04

**Authors:** Philipp Russ, Leo T Wenzel, Simon Bedenbender, Michèle Maeske, Jonas Einloft, Hendrik L Meyer, Andre Ganser, Gert Bange, Martin C Hirsch, Andreas Neubauer, Peter Benoehr, Ivica Grgic

**Affiliations:** Marburg University, Department of Internal Medicine and Nephrology, University Hospital Giessen and Marburg, Marburg, Germany; Marburg University, Institute for Artificial Intelligence in Medicine, University Hospital Giessen and Marburg, Marburg, Germany; Marburg University, Department of Internal Medicine and Nephrology, University Hospital Giessen and Marburg, Marburg, Germany; Marburg University, Department of Internal Medicine and Nephrology, University Hospital Giessen and Marburg, Marburg, Germany; Department of Nephrology, Klinikum Fulda gAG, Marburg University, Campus Fulda, Fulda, Germany; Marburg University, Department of Internal Medicine and Nephrology, University Hospital Giessen and Marburg, Marburg, Germany; Marburg University, Department of Internal Medicine and Nephrology, University Hospital Giessen and Marburg, Marburg, Germany; Marburg University, Department of Internal Medicine and Nephrology, University Hospital Giessen and Marburg, Marburg, Germany; Max-Planck Institute for Terrestrial Microbiology, Marburg, Germany; Marburg University, Center for Synthetic Microbiology (SYNMIKRO) & Departments of Chemistry and Biology, Marburg, Germany; Marburg University, Institute for Artificial Intelligence in Medicine, University Hospital Giessen and Marburg, Marburg, Germany; Marburg University, Department of Internal Medicine, Hematology, Oncology and Immunology, University Hospital Giessen and Marburg, Marburg, Germany; Marburg University, Center for Personalized Medicine, University Hospital Giessen and Marburg, Marburg, Germany; Department of Nephrology, Klinikum Fulda gAG, Marburg University, Campus Fulda, Fulda, Germany; Marburg University, Department of Internal Medicine and Nephrology, University Hospital Giessen and Marburg, Marburg, Germany; Marburg University, Institute for Artificial Intelligence in Medicine, University Hospital Giessen and Marburg, Marburg, Germany

**Keywords:** end stage renal disease, hemodialysis, immersive technology, personalized medicine, virtual reality (VR)

## Abstract

**Background:**

Patients undergoing hemodialysis frequently experience stress, physical discomfort, depressive symptoms and prolonged immobility during lengthy treatment sessions. Immersive virtual reality (VR) has shown promise as a non-pharmacological intervention to improve wellbeing in various clinical settings. However, no multicenter study has examined personalized immersive VR in dialysis patients. This study therefore aimed to assess the tolerability and effects of a single personalized VR session on wellbeing, pain and physiological parameters in patients undergoing hemodialysis.

**Methods:**

In this pre–post single group pilot study, 148 participants from 12 dialysis centers (10 outpatient, 2 in-hospital) were enrolled. Each patient completed one personalized 20-min VR session, selecting from 20 immersive 360° options. Emotional wellbeing and pain were assessed before and after VR exposure, while treatment tolerance, perceived quality and feasibility were assessed post-session. Physiological parameters (blood pressure, heart rate, oxygen saturation) were recorded before, during and after exposure.

**Results:**

Of the 148 enrolled participants, 143 completed the intervention (mean age 62.2 ± 14.5 years; 64.9% male and 35.1% female). Wellbeing improved significantly; among participants reporting pain, scores decreased by ∼50%. Systolic blood pressure declined from 135 to 128 mmHg and diastolic from 72 to 69 mmHg during VR exposure, with heart rate decreasing from a mean of 72 to 67 bpm (*P* < .0001 for all); values returned toward baseline afterwards. No serious adverse events were reported.

**Conclusion:**

Personalized VR was well tolerated and produced measurable psychological and physiological benefits in dialysis patients, supporting its potential as a feasible non-pharmacological adjunct to routine care.

## GRAPHICAL ABSTRACT

**GRAPHICAL ABSTRACT:**
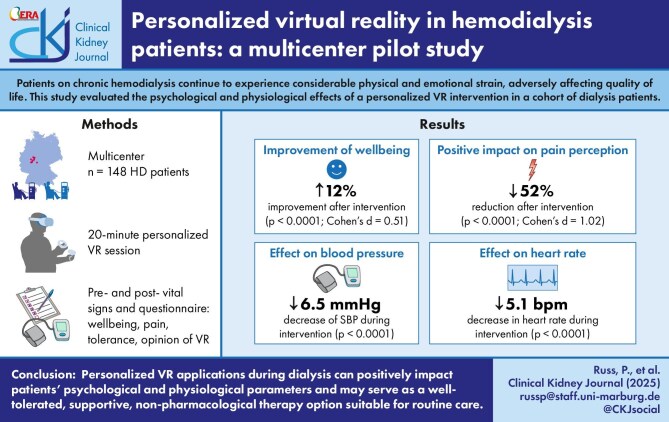

KEY LEARNING POINTS **What was known:**

- Hemodialysis sessions often cause stress, discomfort, monotony and immobility, contributing to depression and reduced quality of life.
- Immersive VR, as a non-pharmacological approach, has shown benefits for both physiological and psychological wellbeing.

**This study adds:**

- No multicenter study has previously explored the effects of personalized immersive VR in dialysis patients.
- A single, tailored session improved subjective wellbeing, reduced pain and induced short-term shifts in cardiovascular parameters, demonstrating feasibility and good patient acceptance.

**Potential impact:**

- Personalized immersive VR is feasible, well tolerated and may provide psychological and physiological benefits in hemodialysis patients.
- It represents a customizable, ‘non-pharmacological’ adjunct that can be easily integrated into routine clinical practice.

## INTRODUCTION

More than 850 million people worldwide are affected by kidney diseases [1]. Among them, approximately 700 million, representing 9%–15% of the global population, are diagnosed with chronic kidney disease (CKD) [2–4]. This patient group is particularly vulnerable due to a higher prevalence of comorbidities and an increased risk of mortality [5]. Within this group, around 5.3 million require renal replacement therapy [4], the majority of whom are dependent on hemodialysis as a life-sustaining therapy, provided it is accessible to them [6]. Despite technological and procedural advancements, many aspects of dialysis therapy remain largely unchanged, as many patients continue to endure substantial physical and psychological burdens [2, 3]. Hemodialysis is typically performed three times per week for 4–5 h, during which patients face immobility, monotony, episodes of fear, frequent machine alarms and discomfort. These factors negatively impact multiple dimensions of health, including subjective wellbeing, sleep quality, cardiovascular health (e.g. blood pressure regulation, heart rate variability and the management of comorbidities), ultimately contributing to a diminished quality of life [7, 8]. Recent research also highlights underrecognized emotional and existential burdens in this population, such as unmet needs for communication around end-of-life care, reflecting a broader gap in patient-centered support [9]. Consequently, the prevalence of depression among hemodialysis patients is estimated to exceed 20% [10]. The global prevalence of CKD continues to rise, and current projections indicate that by 2040, CKD will rank as the fifth leading cause of years of life lost worldwide [11]. Given this trend and the increasing number of patients dependent on hemodialysis, there is a clear need to develop supportive interventions that enhance quality of life and mitigate the physical and psychological burdens associated with long-term dialysis treatment.

Immersive virtual reality (VR) has emerged as a promising non-pharmacological intervention for alleviating anxiety, pain and depression across a variety of medical fields, including psychiatry and psychotherapy, pediatrics, anesthesia and pain management, rehabilitation medicine and critical care medicine [12–17]. Beyond these established areas, a nascent line of research is investigating the use of VR as a therapeutic intervention for hemodialysis patients. Initial findings hold promise, suggesting potential benefits in reducing treatment-associated symptoms and improving patients’ subjective wellbeing [18].

For instance, recent studies have found that VR-enhanced physical activity during dialysis sessions is not only safe, but also significantly improves health-related quality of life among patients [19]. Furthermore, VR interventions have been linked to reduced depressive symptoms and lower inflammatory markers [20, 21]. Complementary pilot studies have applied fully immersive VR for mindfulness-based interventions or in combination with exercise, showing feasibility, safety and promising psychological benefits in small single-center cohorts [22, 23]. Taken together, these findings suggest that beyond supporting physical activity, VR may also confer broader physiological and psychological health benefits. These effects are thought to be mediated by attentional distraction, enhanced presence and embodiment, and neuropsychological changes [24, 25]. One potential pathway is the simulation of natural environments, as these have been shown to promote relaxation and wellbeing [26, 27]. Importantly, many patients on hemodialysis are physically weakened, functionally impaired, medically restricted or otherwise unable to access restorative natural environments that offer such benefits [28, 29]. Immersive VR offers a novel means of providing these patients with access to serene and restorative environments without the need for physical travel.

To the best of our knowledge, no multicenter study has yet investigated the effects of personalized, fully immersive VR exposure in this patient population.

Therefore, the primary aims of this study were:

(i) To evaluate the feasibility and tolerance of a single personalized VR session during routine dialysis.
(ii) To assess effects on patient-reported outcome measures, namely wellbeing and pain [30].
(iii) To examine immediate physiological responses (blood pressure and heart rate).

## MATERIALS AND METHODS

### Participants and study design

**Figure 1:**
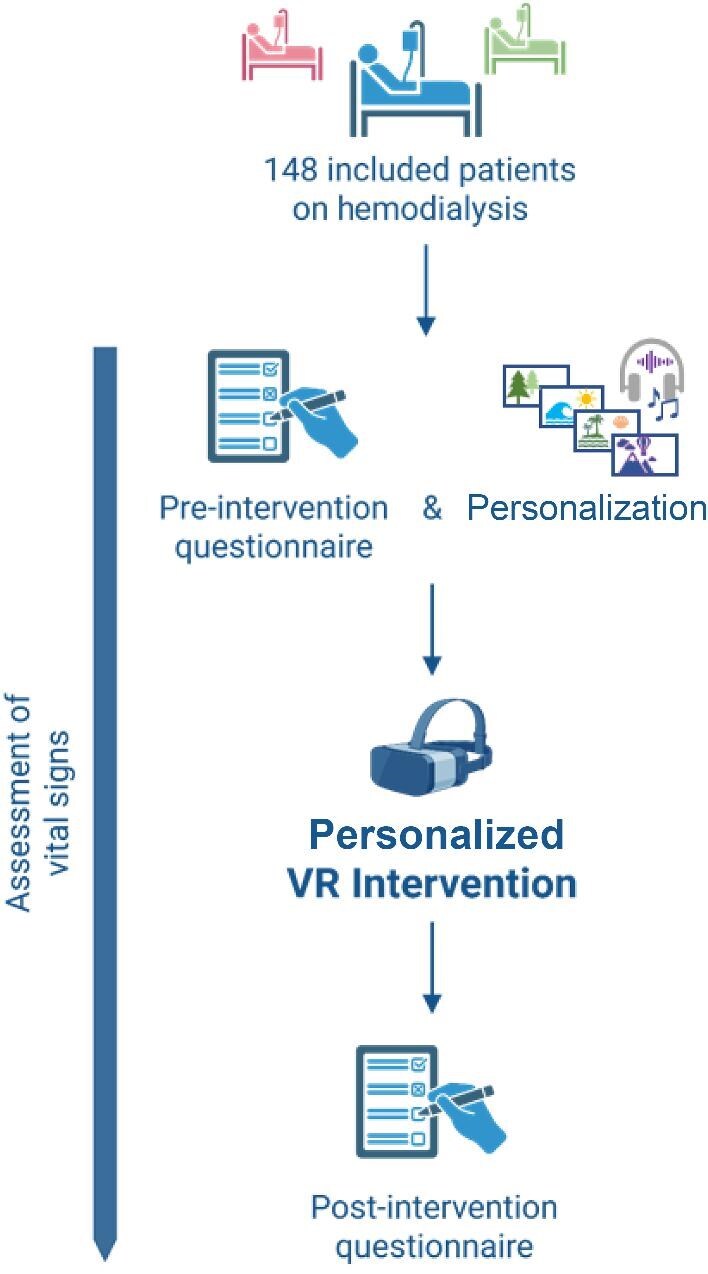
Study flowchart. Overview of enrollment, questionnaires, VR intervention and timing of vital sign assessments. Created in part with BioRender (Bedenbender, S., 2025; https://BioRender.com/dhmmjup).

Between January and March 2025, a total of 148 patients undergoing routine hemodialysis were recruited from 12 German dialysis centers across central Germany, including in Marburg, Fulda, two centers in Giessen, Lich, Kirchhain, Schwalmstadt, Fritzlar, Bad Laasphe, Korbach, and two centers in Wetzlar. Participants were eligible for inclusion if they were aged 18 years or older, undergoing chronic routine dialysis three times per week, and had sufficient cognitive and language abilities, including the ability to read, write and understand spoken instructions. Exclusion criteria comprised a diagnosis of epilepsy, severe visual impairment, advanced dementia, paranoid schizophrenia or colonization with multidrug-resistant bacteria (due to hygiene considerations). Prior to the immersive VR intervention, patients completed a questionnaire and baseline vital parameters (blood pressure, heart rate, oxygen saturation) were recorded. Each participant completed a single 20-min VR session, which was scheduled on different weekdays and not restricted to a specific day. Following the VR experience, patients completed a second questionnaire. Throughout all VR sessions, patient safety and communication were prioritized. A member of the study team remained at the bedside during the entire intervention. An overview of the study design is shown in Fig. 1.

### Study dropouts

Five participants discontinued the VR exposure before completing the full 20-min session. Four of them indicated early that the experience did not meet their personal expectations, while one reported dizziness, prompting termination of the session for safety considerations. Due to incomplete data, these individuals were excluded from both the questionnaire-based and physiological analyses. Only participants who completed the intended duration of the VR intervention were included in the final analysis.

### Immersive VR application

The fully immersive VR intervention was administered using the commercially available Meta Quest 3 head-mounted display (HMD) (Meta Reality Labs, Redmond, Washington USA). This device was selected for its portability, comparatively light weight, wireless functionality and inside-out tracking capabilities, allowing for unrestricted head movement and ease of use in the dialysis setting. To personalize the VR experience, participants could select from 20 immersive 360° audio-visual formats, which were developed and carefully curated by our research team through an iterative design process. These environments included a variety of scenic natural landscapes such as tranquil beaches, picturesque mountains and lush green spaces from different regions of the world, as well as locally inspired settings such as botanical gardens and historic townscapes, and contemplative indoor locations like church interiors.

**Figure 2:**
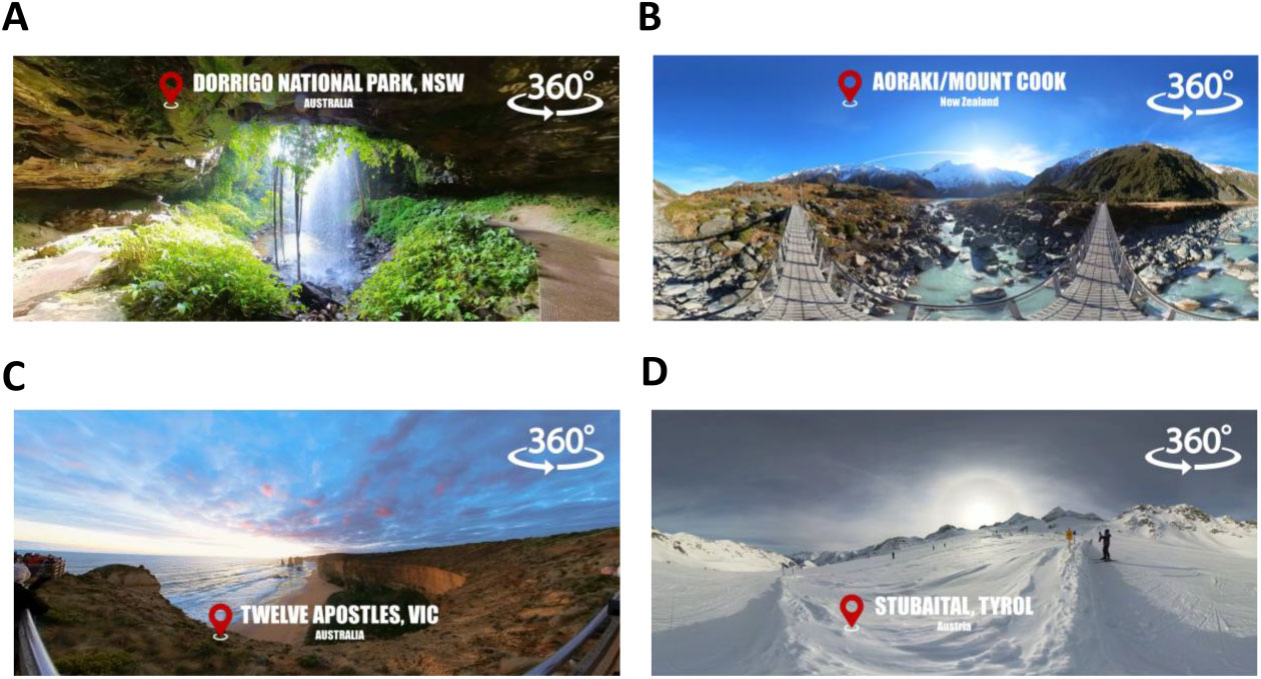
Representative VR environments. Examples of curated 360° immersive landscapes available to participants, including (**A**) forest, (**B**) mountain, (**C**) coastal and (**D**) alpine settings.

Background audio was also customizable, with options including nature sounds, classical music and ambient melodies. Each curated VR experience lasted approximately 20 min and included scene transitions every 2–3 min to maintain visual engagement. To optimize immersion and minimize external distractions, such as sudden dialysis machine alarms, participants wore headphones with moderate noise cancellation throughout the intervention. The level of sound isolation was limited, allowing patients to maintain verbal communication with dialysis staff at all times. In addition, a member of the study team was present at the bedside throughout the intervention to ensure continuous supervision and patient safety. Following each use, all devices were cleaned and disinfected in accordance with institutional hygiene protocols. The VR sessions were applied during the first half of the dialysis treatment, typically within the first 120 min after initiation. Representative snapshots of the immersive 360° environments are presented in Fig. 2, and a comprehensive list of all available formats and customization options is provided in Supplementary data, Table S1.

### Questionnaire design, data acquisition and analysis

The questionnaire included open-ended, closed-ended and numerically scaled questions, comprising 35 items on demographics, medical background, prior VR experience, expectations and perceptions of the intervention. Pain and wellbeing were assessed both before and after the VR intervention using 0–10 numerical rating scales. Post-intervention questions addressed perceived video and audio quality, sense of presence and perspectives on future use, including willingness to use VR repeatedly during dialysis sessions (all 0–10 scales or categorical items). Simulator sickness was evaluated once, immediately after the VR intervention, using the validated Simulator Sickness Questionnaire (SSQ) consisting of 16 Likert-scale items [31]. Physiological parameters (heart rate, blood pressure and oxygen saturation) were measured at three fixed, non-continuous time points (immediately before VR, at minute 15 of the intervention, and 10 min after completion). Blood pressure was assessed using certified electronic upper-arm devices, and heart rate and oxygen saturation with certified pulse oximeters. Measurements were standardized across all sites and performed exclusively by trained members of the core study team. A full overview of all questionnaire items and recorded parameters is provided in Supplementary data, Table S2.

All responses and recorded parameters were entered into Microsoft Excel (version 16, Redmond, WA, USA) and served as the basis for subsequent analyses. Statistical evaluations and graphical representations were conducted using GraphPad Prism (version 10; Boston, MA, USA), R (version 4.5.0; Vienna, Austria) and RStudio (version 2024.12.1; Boston, MA, USA). The study design flowchart (Fig. 1) and in part the graphical abstract were created using BioRender (Toronto, Canada). Mean values, standard deviations (SD) and medians with interquartile ranges (IQR) were calculated to describe central tendencies and data variability. Results are reported as mean ± SD and, where appropriate, as median with IQR. Statistical differences between pre- and post-intervention values were assessed using paired *t*-tests. Effect sizes were calculated using Cohen’s *d*. Statistical significance was defined as α ≤ 0.05, with significance levels indicated as follows: *P ≤ .05, **P < .01, ***P < .001 and ****P < .0001.

### Ethics approval and trial registration

The study was approved by the Ethics Committee of the Medical Faculty at Philipps University of Marburg (file no. 23–223 BO) and registered with the German Clinical Trials Register (registration no. DRKS00036521). The study followed the Declaration of Helsinki and local ethical guidelines for research involving human subjects.

## RESULTS

### Baseline characteristics

A total of 148 patients from 12 dialysis centers were enrolled in the study. The sample consisted of 96 males (64.9%) and 52 females (35.1%), with a mean age of 62.2 years (SD ± 14.5; range: 21–90 years). Of the 148 participants, 143 completed the full immersive VR session, while five discontinued early. VR sessions were distributed across weekdays as follows: Monday (*n* = 37), Tuesday (*n* = 9), Wednesday (*n* = 54), Thursday (*n* = 11), Friday (*n* = 28) and Saturday (*n* = 4). Approximately one-quarter of participants had prior experience with VR, and one reported using a headset on a weekly basis. Notably, the most frequently selected landscapes featured natural environments such as coastlines, beaches and mountains. Regarding preferences for the accompanying audio, 64 participants (43.2%) chose ambient music, 58 (39.2%) selected the original video sound (consisting solely of nature sounds captured during recording) and 26 (17.6%) opted for classical music. Detailed baseline characteristics are summarized in Table 1.

**Table 1:** Demographic characteristics of the entire cohort.

Participants-No.%		
Total	148	100
Completion	143	96.6
Dropouts	5	3.4
Sex		
Female	52	35.1
Male	96	64.9
Age—years		
Mean (SD)	62.2 (14.5)	
Min < Median < Max	21 < 64.5 < 90	
Dialysis center		
Hospital of Fulda	12	8.1
Marburg-Cappel	18	12.2
Kirchhain	18	12.2
Giessen (Doctor’s practice)	15	10.1
Giessen (University hospital)	10	6.8
Lich	8	5.4
Schwalmstadt	5	3.4
Fritzlar	9	6.1
Wetzlar I	17	11.5
Wetzlar II	12	8.1
Bad Laasphe	15	10.1
Korbach	9	6.1
Chosen video		
Australia Great Ocean Road	36	24.3
Botanical Garden Marburg	10	6.8
Church	1	0.7
Church Concert	1	0.7
City of Marburg I	15	10.1
City of Marburg II	2	1.4
Croatia Harbour	2	1.4
Dorrigo National Park (Rainforest)	1	0.7
Forest Hesse	1	0.7
New Zealand I (Mount Cook)	4	2.7
New Zealand II (Prairie and green hills)	4	2.7
San Diego	12	8.1
Sydney	5	3.4
Thailand	28	18.9
Tyrol	26	17.6
Chosen sound		
Original	58	39.2
Classical music	26	17.6
Ambient sound music	64	43.2
Previous experience with VR		
Yes	39	26.4
No	109	73.6

Overview of participant demographics, dialysis center distribution, selected VR content and prior VR experience.

min, minimum; max, maximum.

### Improvement in wellbeing and pain

**Figure 3:**
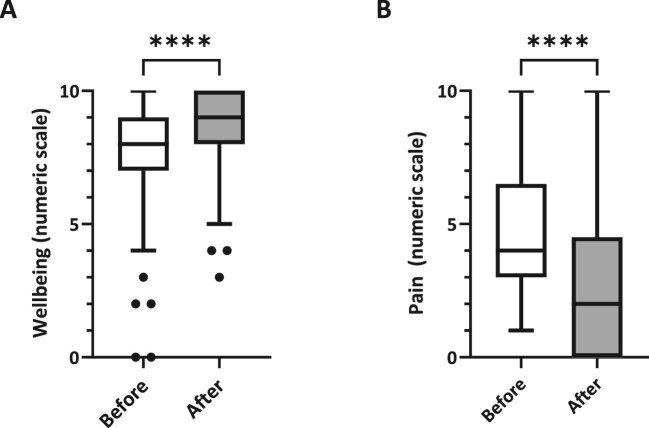
Patient-reported outcome. (**A**) Boxplots of self-reported wellbeing scores before and after VR. (**B**) Boxplots of pain scores in the pain subgroup (*n* = 25) before and after VR. Boxes show median and IQR; whiskers indicate range.

Following the intervention, self-reported wellbeing significantly increased by about 11.7% from a mean of 7.7 (SD ± 1.9; IQR 7–9) to 8.6 (SD ± 1.5; IQR 8–10; *P* < .0001), which corresponds to a medium effect size (Cohen’s *d* = 0.51; Fig. 3A).

Of all participants, 25 participants reported experiencing pain. In this subgroup, the average pain score decreased significantly by approximately 52.1% from 4.8 (SD ± 2.4; IQR 3–6.5) before the intervention to 2.3 (SD ± 2.7; IQR 0–4.5) afterward (*P* < .0001), indicating a large effect size (Cohen’s *d* = 1.02; Fig. 3B).

### Impact on cardiovascular parameters

**Figure 4:**
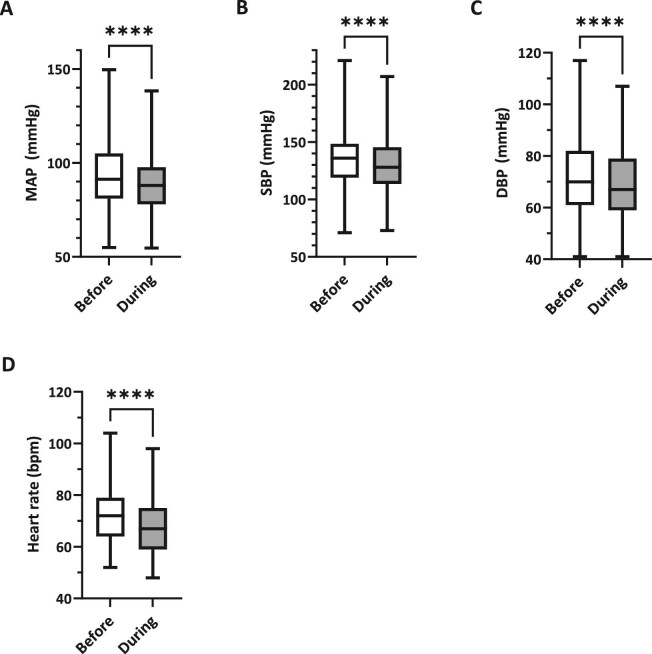
Cardiovascular parameters pre-VR vs during VR. Boxplots comparing (**A**) MAP, (**B**) SBP, (**C**) DBP and (**D**) heart rate measured immediately before VR and at minute 15 during VR. Boxes show the median and IQR; whiskers indicate the minimum to maximum range.

During personalized VR intervention, mean arterial pressure (MAP) significantly decreased from a pre-intervention mean of 93.1 millimeters of mercury (mmHg) (SD ± 17.5; IQR 81.0–105.0) to 89.2 mmHg (SD ± 16.7; IQR 78.0–97.7) toward the end of the VR exposure (*P* < .0001; Fig. 4A). Systolic blood pressure (SBP) showed a comparable reduction, declining from an average of 134.9 mmHg (SD ± 26.0; IQR 119.0–148.5) to 128.4 mmHg (SD ± 24.5; IQR 113.5–145.4; *P* < .0001; Fig. 4B) by the end of the exposure. Diastolic blood pressure (DBP) also showed a statistically significant decrease from 71.8 mmHg (SD ± 15.7; IQR 61.0–82.0) to 69.2 mmHg (SD ± 15.1; IQR 59.0–79.0; *P* < .0001; Fig. 4C). Effect sizes were small with Cohen’s *d* values of –0.23 for MAP, –0.26 for SBP and –0.17 for DBP. Notably, BP values returned to near-baseline levels 10 min after the intervention. Ten minutes after the intervention, MAP was 92.7 mmHg (SD ± 17.0), which was not significantly different from the pre-intervention value of 93.1 mmHg (*P* = .29; *n* = 110).

Heart rate also decreased significantly, from a pre-intervention average of 72.4 beats per minute (bpm) (SD ± 11.3; IQR 64.0–79.0) to 67.3 bpm (SD 10.5; IQR 59.0–75.0) by the end of the intervention (*P* < .0001), corresponding to a medium effect size (Cohen’s *d* = –0.47; Fig. 4D). Ten minutes after the intervention, heart rate had increased to 69.1 bpm (SD ± 10.6; IQR 61.8–75.3) but remained significantly lower than baseline (*P* = .0003; *n* = 110).

### Subgroup analysis by prior VR experience

In a subgroup analysis stratified by prior VR experience, no significant differences were observed between VR naïve (*n* = 108) and VR non-naïve participants (*n* = 35) in improvements in wellbeing (*P* = .48), reductions in pain (*P* = .56), systolic blood pressure (*P* = .85) or heart rate (*P* = .24). These results are shown in Supplementary data, Fig. S1.

### Assessment of quality, personalization and usability

Participants rated the overall quality of the personalized immersive VR formats including the accompanying acoustics. On average, the 360º visual aspects received an overall quality rating score of 7.9 (SD ± 1.9), while the audio aspect was rated with a mean score of 7.6 (SD ± 2.1). Participants rated the importance of personalization features, specifically the ability to choose individual 360º virtual environments and accompanying sound experiences. These customization options were highly valued: the option to select virtual environments received a mean rating of 8.0 (SD ± 2.2), while the ability to choose the audio was rated at 8.2 (SD ± 1.9). These findings highlight a strong user preference for personalized and controllable immersive experiences.

System usability was also rated positively, with an average score of 8.0 (SD ± 1.7), indicating a generally favorable user experience. A detailed summary of these evaluation results is presented in Supplementary data, Fig. S2A.

### Willingness to use and tolerance of VR during dialysis

Fifteen patients (10.5%) reported that they were not inclined to use immersive VR during dialysis. In contrast, 127 patients (88.8%) were inclined to use VR at least once per week (*n* = 1 missing). Among them, 51 (35.7%) preferred using VR during every session, 48 (33.6%) once per week and 28 (19.6%) twice per week (Supplementary data, Fig. S2B).

**Figure 5:**
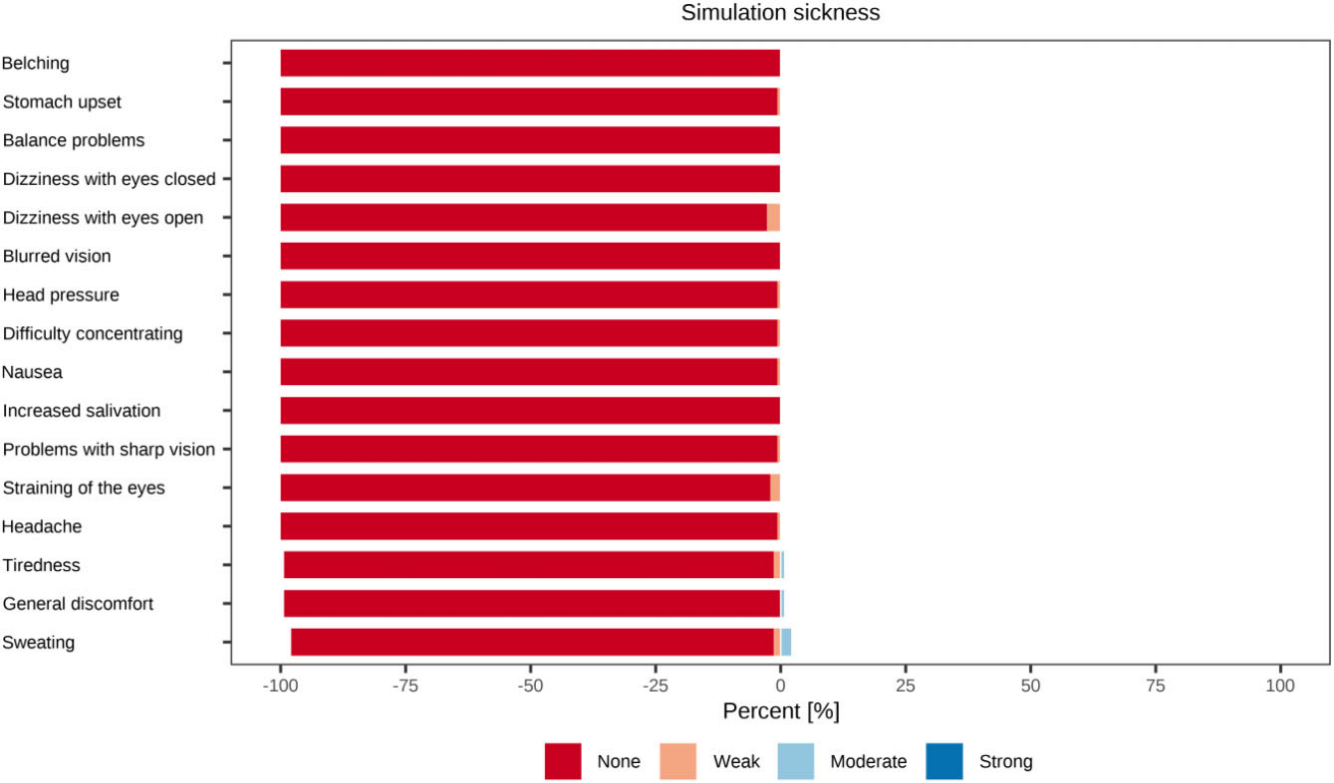
Tolerability of VR intervention. Distribution of simulator sickness symptoms reported after VR, categorized by severity (none, weak, moderate, strong).

According to the SSQ, the VR intervention was very well tolerated. The mean total severity score was 0.7 (SD ± 3.4), with mean subscale scores of 0.9 (nausea), 0.6 (oculomotor) and 0.7 (disorientation). A total of 131/143 participants (91.6%) reported no symptoms; among the remaining 12, only mild symptoms were present, most commonly sweating (3.5%) and dizziness with eyes open (2.8%). These very low scores likely reflect the mostly passive nature of the VR exposure during dialysis, which required no enhanced or active head or body movement. An overview of the SSQ results is provided in Fig. 5.

## DISCUSSION

This multicenter pilot study demonstrates that personalized immersive VR is a valuable and well-accepted adjunct to standard care in a heterogeneous cohort of dialysis patients. Beyond significantly reducing perceived pain and enhancing subjective wellbeing, VR immersion also had measurable effects on physiological parameters. Moreover, the positive patient feedback underscores the demand for alternative, non-pharmacological supportive approaches during dialysis, since patients with advanced CKD frequently experience multiple burdensome symptoms, while evidence for effective non-pharmacological management remains limited [32].

Our findings are consistent with prior studies in related contexts. For example, one study with a total of 26 participants reported that exposure to virtual forest landscapes for 6 min per session over a 3-week period led to a significant reduction in heart rate and elicited more positive emotional responses [26]. Similarly, the analgesic potential of VR has been demonstrated in several studies, with reported reductions in pain across various clinical contexts [33], and its use in pain management is becoming more recognized [14, 34]. Taken together, the emerging evidence suggests that immersive VR may serve as a valuable complementary approach in the care of dialysis patients.

In our study, a modest decrease in blood pressure was observed during the VR intervention. While this decline may have been partially attributable to ultrafiltration, blood pressure values returned to baseline within a short period after the session. Heart rate likewise decreased during the intervention, exhibited a mild rebound soon after, yet remained slightly below baseline. Taken together, these findings suggest that the observed shifts in cardiovascular parameters during VR exposure were transient and largely reversible. The rapid return of blood pressure to baseline, despite ongoing ultrafiltration, indicates that observed effects were unlikely to be solely due to fluid removal and may have been modulated by the immersive experience itself. This aligns with evidence suggesting that, compared with conventional, non-immersive distractions (e.g. TV or radio), immersive VR provides a more multisensory and engaging environment that may more effectively engage autonomic regulation. Studies have shown that immersive VR environments can reduce sympathetic arousal and enhance parasympathetic activity compared with passive or less immersive conditions [35, 36]. However, since this study did not directly compare VR with other forms of attentional diversion, further controlled research is required to evaluate its relative efficacy.

The mechanisms underlying the observed physiological and psychological effects of immersive VR are likely multifactorial. While attentional distraction from treatment-related discomfort is a well-established pathway [12, 37], distraction alone does not fully explain the observed benefits [24]. VR may also engage affective and motivational processes and has been associated with neurophysiological changes, for example through mechanism of exposure and conditioning [24, 38–40]. Importantly, VR interventions have also been shown to strengthen pain self-efficacy and enhance coping behavior, suggesting an increased sense of control in challenging clinical contexts [41, 42]. Furthermore, immersive VR enhances the sense of presence and embodiment, which has been shown to correlate with stronger analgesic effects [25]. Beyond distraction and neuropsychological mechanisms, VR environments that simulate natural settings may also recreate the calming, restorative effects typically associated with real-world exposure to nature. Indeed, nature-based interventions, including forest bathing, have been shown to reduce blood pressure, heart rate, anxiety and stress, while improving mood and mental clarity [43]. Yet, many dialysis patients are physically unable to access such environments due to mobility limitations, treatment demands or comorbid conditions [28, 29]. VR addresses this gap by enabling virtual access to these soothing settings during dialysis sessions. Moreover, as VR technology continues to advance, its potential to enrich the lived experience of patients undergoing routine medical procedures becomes increasingly evident [44, 45].

In terms of tolerability, more than 90% of participants reported no simulator sickness at all, and the few reported symptoms were uniformly mild, resulting in mean SSQ scores close to zero. Comparable findings have been reported in a study using passive 360° video VR in older adults, where SSQ scores were also very low [46]. Our scores were even lower, which is likely attributable to the highly passive setting of hemodialysis, where patients remained in a recumbent position without active head or body movement.

Feasibility was likewise supported by patient feedback. The vast majority of participants rated the video and audio quality highly and reported a strong sense of presence during the VR session. Importantly, most patients indicated willingness to use VR repeatedly during future dialysis treatments, underscoring its acceptability and potential for sustainable integration into routine care.

In addition to tolerability and feasibility, safety and communication also require consideration in the dialysis setting. Although patients used headphones, the level of sound isolation was moderate and did not preclude verbal communication with staff. Furthermore, a member of the study team was present at bedside throughout all sessions, and no restrictions or limitations in dialysis treatment were reported. Nonetheless, for future integration into routine dialysis care, the use of non-noise-cancelling headphones and staff training to ensure continuous communication with patients are recommended to maximize safety.

Despite these promising results, several limitations must be acknowledged. The observed benefits may partly reflect a novelty effect from first VR exposure. Moreover, only short-term outcomes were assessed, as no long-term follow-up was conducted. Furthermore, the absence of a control group precludes causal inference. Symptom burden may also vary across the week: the first dialysis session after the long interdialytic interval is often associated with greater fluid overload and more pronounced symptoms [47], although other research did not find significant day-of-week differences in symptom severity [48]. Our exploratory analyses likewise revealed no consistent differences between early- and late-week sessions, although fluid removal itself may still have contributed to the observed changes. In addition, reliance on self-reported measures may introduce bias, particularly in symptom and preference assessments, and the use of a non-validated, study-specific questionnaire further restricts interpretability. Finally, despite multicenter recruitment, the sample may not fully represent the broader dialysis population. Individuals who chose to engage with VR may differ systematically from those who declined participation, introducing potential selection bias. Future randomized controlled trials with longitudinal follow-up are required to validate and extend these preliminary findings.

## CONCLUSION

This multicenter study demonstrates that fully immersive, personalized VR is well accepted and generally well tolerated among a heterogeneous group of patients receiving chronic hemodialysis. A single, personalized session produced meaningful short-term benefits, including reductions in pain perception, blood pressure and heart rate. The strong preference for personalization and the absence of notable adverse effects highlight its feasibility as a supportive, patient-centered intervention. Given its ease of integration into existing treatment routines, immersive VR may represent a promising and potentially cost-effective digital therapeutic to enhance quality of life in this vulnerable population. Nonetheless, important limitations, including the absence of a control group, reliance on self-reported measures, and potential selection bias, must be acknowledged. These findings should therefore be viewed as preliminary but promising, laying the groundwork for future randomized controlled trials with longitudinal follow-up and repeated VR sessions. Such studies, benchmarked against usual care, are required to rigorously evaluate the therapeutic efficacy and long-term impact of immersive VR in clinical nephrology settings.

## Supplementary Material

sfaf367_Supplemental_File

## Data Availability

All relevant data is reported in the article. Additional data can be provided by the corresponding author on reasonable request.
