# Supplementary material for: Personalized virtual reality in hemodialysis patients: a multicenter pilot study
Source: Clin Kidney J. 2025 Dec 4;19(1):sfaf367. doi: 10.1093/ckj/sfaf367 (PMC12757744; doi:10.1093/ckj/sfaf367)
Supplement: sfaf367_Supplemental_File [file sfaf367_supplemental_file.docx]

Supplemental Material

***“*Personalised Virtual Reality in Haemodialysis Patients: A Multicentre Pilot Study*”***

Supplemental Table 1 | List of available 360° videos.

| **Video category** | **Video title** |
| --- | --- |
| **Beach landscapes** | Australia Great Ocean Road |
|  | Thailand |
|  | San Diego |
|  | Coast of Istria (Croatia) |
| **City landscapes** | Sydney |
|  | Various cities of Croatia |
|  | Croatia Harbour |
| **Forest landscapes** | Forest of Hesse |
|  | Dorrigo National Park (Rainforest) |
| **Local environments** | City of Marburg I (Old Town) |
|  | City of Marburg II (Southern City and river landscape) |
|  | Botanical Garden of Marburg I |
|  | Botanical Garden of Marburg II |
|  | Coffee house/Bar in Marburg |
| **Mountain landscapes** | Tyrol |
|  | New Zealand I (Mount Cook) |
| **Church** | Church |
|  | Church concert |
| **Others** | New Zealand II (Prairie and green hills) |
|  | Various landscapes of Croatia |

Supplemental Table 2 | Design of the questionnaire.

| **Question (Number)** | **Question type** | **Question text** |
| --- | --- | --- |
| Demographics | Open  Single choice  Open  Open  Open  Open | Age:  Gender:  Location:  Underlying renal disease:  Beginning of dialysis:  Vascular access (e.g., Shunt) |
| 1 | Binary (yes or no) | **Questions before VR application**  Have you ever tried VR glasses? |
| 2 | Binary (yes or no) | If yes, do you use them frequently (more than once a week)? |
| 3 | Numeric scale (0 to 10 scale) | What is your level of wellbeing at the moment? |
| 4 | Numeric scale (0 to 10 scale) | How much do you expect the VR video to have a positive effect on your mood? |
| 5 | Numeric scale (0 to 10 scale) | How much do you expect the VR video to have a positive effect on your wellbeing? |
| 6 | Numeric scale (0 to 10 scale) | Do you have any pain? |
| 7 | Numeric scale (0 to 10 scale) | Do you think the video could relieve pain? |
| 8 | Numeric scale (0 to 10 scale) | Do you think the video can positively distract you from the dialysis environment? |
| 9 | Open | What do you expect from the video? |
| 10 | Numeric scale (0 to 10 scale) | **Questions after VR application**  What is your level of wellbeing at the moment? |
| 11 | Numeric scale (0 to 10 scale) | How would you rate the overall quality of the video? |
| 12 | Numeric scale (0 to 10 scale) | The option to choose the video type is a valuable feature. |
| 13 | Numeric scale (0 to 10 scale) | How would you rate the usability of the VR glasses? |
| 14 | Numeric scale (0 to 10 scale) | How intense was your sense of 'presence', of actually being in/at the location of the video? |
| 15 | Numeric scale (0 to 10 scale) | How did you like the background sound? |
| 16 | Numeric scale (0 to 10 scale) | The option to choose the background music is a valuable feature. |
| 17 | Numeric scale (0 to 10 scale) | Did you feel relaxed after watching the VR video? |
| 18 | Numeric scale (0 to 10 scale) | If you could use the VR glasses more often during dialysis, would you? |
| 19 | Single choice (4 points) | How often would you wear the VR glasses on dialysis? |
| 20 | Single choice (6 points) | How long would you wear VR glasses during dialysis? |
| 21 | Numeric scale (0 to 10 scale) | Would you look forward to your next dialysis session if you knew you could wear VR glasses and watch videos? |
| 22 | Numeric scale (0 to 10 scale) | Would you like to wear VR glasses at every dialysis session in the future? |
| 23 | Numeric scale (0 to 10 scale) | Do you have any pain? |
| 24 | Numeric scale (0 to 10 scale) | Do you think the video could relieve pain? |
| 25 | Numeric scale (0 to 10 scale) | How much do you expect the VR video to have a positive effect on your wellbeing? |
| 26 | Numeric scale (0 to 10 scale) | How much do you expect the VR video to have a positive effect on your mood? |
| 27 | Open | What was your overall impression of the VR video? Are there any aspects that struck you as particularly positive or negative? |
| 28 | Open | Could you imagine wearing VR glasses outside of dialysis? If so, where? |
| 29 | Binary (yes or no) | Did you experience any side effects from the VR application? |

| **Simulation Sickness Questionnaire** | Likert (4 point) | *For every symptom, please state in which intensity it appears in this moment:*  General discomfort  Tiredness  Headache  Straining of the eyes  Problems with sharp vision  Increased salivation  Sweating  Nausea  Difficulty concentrating  Head pressure  Blurred vision  Dizziness with eyes open  Dizziness with eyes closed  Balance problems  Stomach upset  Belching |  |
| --- | --- | --- | --- |

VR, virtual reality.


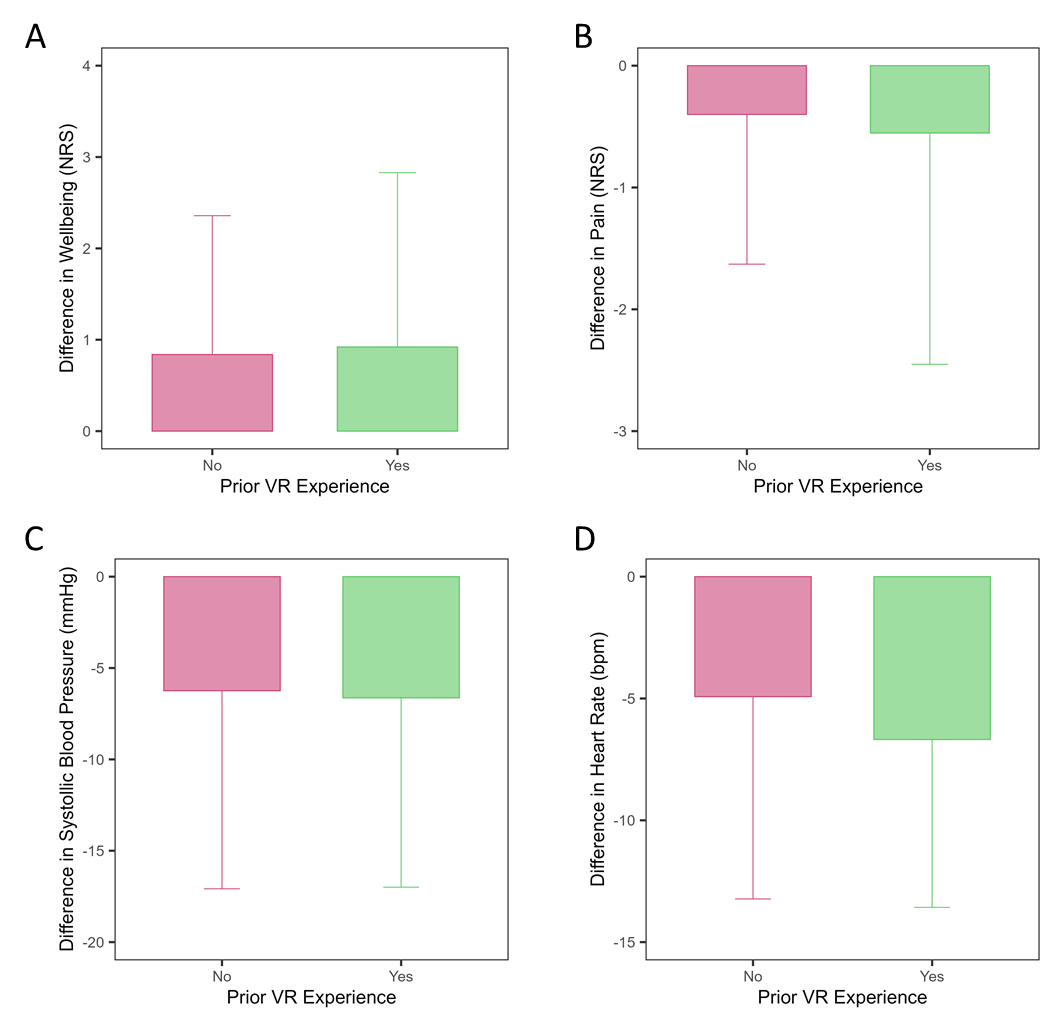


**Supplemental Figure 1 | Subgroup analysis by prior VR experience.** Boxplots of changes (post–pre difference) in (A) wellbeing, (B) pain, (C) systolic blood pressure, and (D) heart rate, stratified by participants without (No) and with (Yes) prior VR experience. Boxes show median and IQR; whiskers indicate range.


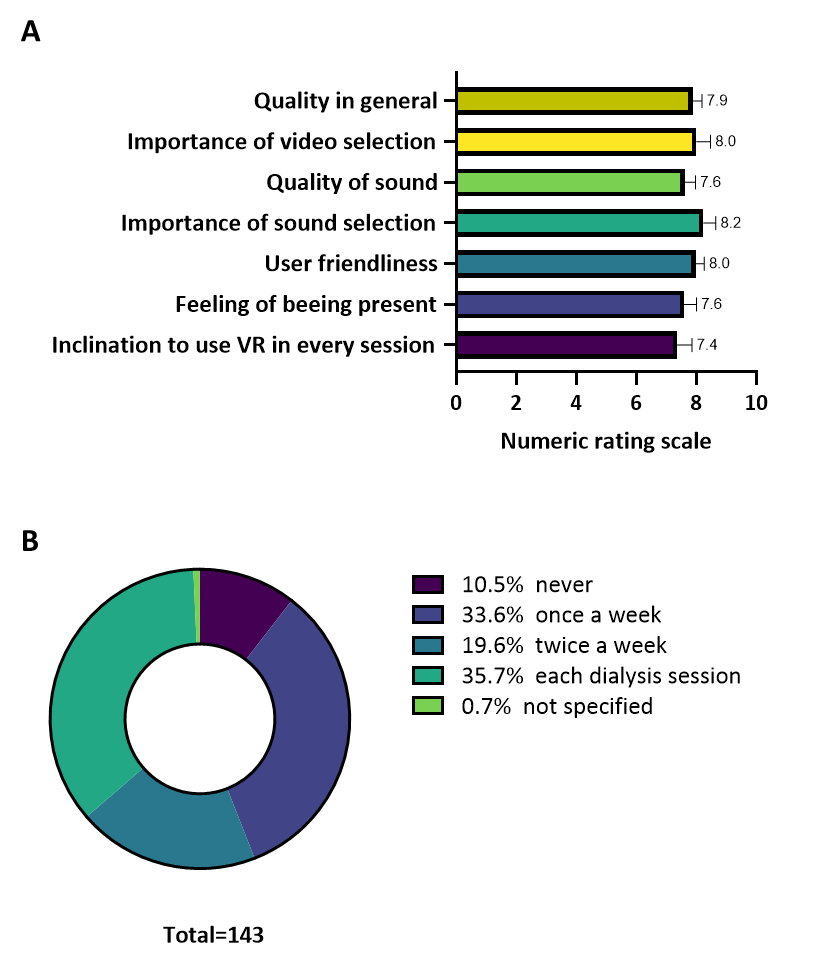


**Supplemental Figure 2 | Evaluation of VR experience and willingness to use.** (A) Ratings of visual quality, audio quality, personalization options, and usability on a 0–10 scale (higher = better). (B) Intended frequency of VR use during dialysis sessions (proportion of participants per category).
